# Systematic review of health related-quality of life in adults with osteogenesis imperfecta

**DOI:** 10.1186/s13023-023-02643-3

**Published:** 2023-02-22

**Authors:** Darran Mc Donald, Tara Mc Donnell, Julie Martin-Grace, Gerry Mc Manus, Rachel K. Crowley

**Affiliations:** 1 Department of Endocrinology, St Vincent’s University Hospital, Dublin, Ireland; 2 School of Medicine, University College Dublin, Dublin, Ireland; 3 Department of Informatics, St Vincent’s University Hospital, Dublin, Ireland; 4 Academic Department of Endocrinology, Beaumont Hospital, Dublin, Ireland; 5 Rare Disease Clinical Trial Network, Dublin, Ireland

**Keywords:** Osteogenesis imperfecta, Quality of life, Health-related quality of life, Adult, Burden of disease, Activities of daily life, Health status, SF-36, Physical component score, Mental component score

## Abstract

**Background:**

Osteogenesis imperfecta (OI) is a rare, connective tissue disorder characterised by bone fragility, resulting in recurrent fractures and skeletal deformities. Extra-skeletal manifestations include dentinogenesis imperfecta, hearing abnormalities and lung disease. These co-morbidities combined with recurrent fractures can exert a significant impact on health-related quality of life (HR-QOL). It is important to assess HR-QOL throughout adulthood because the prevalence of some OI-specific complications increases with age.

**Methods:**

PubMed, EMBASE and CENTRAL databases were searched on 2nd February 2022 to identify studies reporting quantitative assessments of HR-QOL in adults with OI. The primary endpoint was to determine the impact of an OI diagnosis on adult’s HR-QOL. Secondary endpoints were to (i) examine how frequently various HR-QOL assessment tools were used (ii) identify differences in HR-QOL between OI types and (iii) investigate the determinants of HR-QOL in adults with OI. Search results were exported to Endnote where two reviewers independently conducted title/abstract and full-text reviews. Data from accepted studies were extracted into Microsoft Excel. A narrative synthesis was then undertaken.

**Results:**

The review identified 17 studies with a total of 1,648 adults. The Short Form-36 (SF-36) was the most frequently reported HR-QOL assessment tool and was used in nine studies. Physical HR-QOL was reduced in adults with OI. Physical component scores (PCS) or individual physical domains of the SF-36 were lower in eight of nine studies. Mental component scores (MCS) were preserved in all six studies, however individual mental health domains of the SF-36 were reduced in some studies. The prevalence of anxiety/depression was relatively low in adults with OI. Those with type III OI had lower physical and respiratory HR-QOL but preserved mental HR-QOL compared with type I. The prevalence of fatigue and pain was higher in adults with OI compared with reference populations. Age and cardio-pulmonary co-morbidities were associated with lower HR-QOL.

**Conclusion:**

OI in adulthood has a wide-ranging negative impact on HR-QOL. Physical and respiratory HR-QOL were lower, while the prevalence of pain and fatigue were higher than in reference populations. Mental HR-QOL was relatively preserved, although some deficits were identified. Age and cardio-pulmonary co-morbidities were associated with lower HR-QOL.

**Supplementary Information:**

The online version contains supplementary material available at 10.1186/s13023-023-02643-3.

## Background

Osteogenesis imperfecta (OI) is a rare, connective tissue disorder characterised by low bone mineral density and recurrent fractures. The disorder has a prevalence of 1 to 2 per 10,000 [1]. The original Sillence classification designated OI by clinical severity with four subtypes; type I-mild (ORPHA: 216,796), type II-neonatally lethal (ORPHA: 216,804), type III-severe (ORPHA: 216,812) and type IV-moderately severe (ORPHA: 216,820) [2]. The four subtypes recognised in this classification are caused by mutations in the *COL1A1* and *COL1A2* genes, which encode the alpha-1 and alpha-2 chains of type 1 collagen with an autosomal dominant pattern of inheritance and account for 90% of OI cases [3, 4]. Mutations in these genes result in reduced synthesis or altered structure of type 1 collagen. The current classification of OI includes 18 subtypes, the remaining types are largely caused by mutations in genes that regulate bone mineralisation (types V, VI), post-translational modification (types VII–IX), processing and crosslinking (types X–XII) of type 1 collagen, in addition to osteoblast differentiation and bone mineralisation (types XIII–XVIII) with autosomal recessive and X-linked patterns of inheritance [5, 6].

OI is a heterogenous disorder with significant variation in clinical features and severity. Type I OI is characterised by non-deforming fractures, which result from minor trauma and primarily occur in childhood and adolescence. Individuals with type I OI typically achieve normal height and can mobilise independently. In contrast, those with type III suffer in utero fractures and experience progressive lower limb and spinal deformities, resulting in short stature and an inability to bear weight or mobilise independently [7]. Dentinogenesis imperfecta, craniofacial abnormalities and joint hypermobility are additional musculoskeletal features of OI. Extra-skeletal manifestations include blue sclerae, hearing impairment, skin bruising and pulmonary and cardiovascular abnormalities [8]. Intrinsic and extrinsic lung disease is common in OI and represents a significant source of morbidity and mortality [9]. Common cardiovascular manifestations include valvular defects such as aortic and mitral regurgitation and atrial fibrillation. The combination of recurrent fractures and multiple co-morbidities has the potential to exert a significant negative impact on individuals’ health-related quality of life (HR-QOL). Although there is no cure for OI, management centres around physical therapy to maintain function, pharmacological therapy to increase bone mineral density and orthopaedic surgeries to treat fractures, stabilise long bones and prevent deformities [10, 11]. The medical management of OI requires a specialist multidisciplinary team approach with the aim of promoting physical function, mental health, independence and ultimately HR-QOL.

HR-QOL refers to an individual’s perception of their physical, psychological and social well-being [12]. HR-QOL assessments in the clinical setting can facilitate shared decision-making, identify individuals with impaired HR-QOL that are amenable to intervention, and evaluate the impact of treatments [13, 14]. There is no consensus on which HR-QOL assessment tool should be used in OI at present. Multiple studies have reported HR-QOL outcomes in children, adolescents and their caregivers, giving us a clearer understanding of the burden of disease in a younger cohort [15–17]. HR-QOL in adults with OI has received less attention, despite the condition having the potential to exert a greater impact with increasing age. For instance, osteoarthritis and cardio-respiratory complications increase with advancing age. It is therefore important to have a clear understanding of the impact of OI throughout an individual’s life.

Dahan-Oliel et al. performed a systematic review in 2015 which reported that OI was associated with reduced physical but preserved mental HR-QOL [18]. However, most studies in the Dahan-Oliel review assessed HR-QOL in children. Several studies evaluating HR-QOL of adults with OI have been published in the intervening years, which has significantly expanded the literature in this area. Therefore, there is a need to collate the data on the burden of OI in adulthood. This systematic review aimed to evaluate HR-QOL in adults with OI. We also intended to determine which assessments are used to assess HR-QOL, identify any differences in HR-QOL between types of OI and describe the determinants of HR-QOL in OI.

## Methods

A systematic review of the literature was performed by following the 2020 preferred reporting items for systematic reviews and meta-analyses (PRISMA) guidelines [19]. The review was registered with PROSPERO before formal searches were performed (ID CRD42022295164).

### Primary and secondary endpoints

The primary endpoint of the review was to determine the impact of an OI diagnosis on adult HR-QOL. The secondary endpoints were to (i) examine how frequently various HR-QOL assessment tools were used; (ii) identify differences in HR-QOL between types of OI, and (iii) investigate the determinants of HR-QOL in adults with OI.

### Search strategy and study selection

A systematic literature search of PubMed, EMBASE and CENTRAL databases was conducted. The search strategy was created in consultation with an information specialist. The terms ‘osteogenesis imperfecta’ AND 'quality of life' OR 'activit* of daily living' OR 'quality adjusted life year*' OR 'health status' OR 'daily life activity' OR 'health status’ were searched for all papers from 1st of January 1978 to the 2nd of February 2022. Index terms were used where available (e.g. MESH terms in PubMed). Detailed documentation of the search process was recorded in a search log which is available in Additional file 1. Studies were exported to Endnote and duplicate articles removed. Two reviewers screened the title and abstracts of papers identified by the literature search independently. They then screened the full-texts of potentially relevant articles to confirm their inclusion in the review. At each stage, in the event of a disagreement, reviewers had a discussion, followed by adjudication from the senior author if consensus could not be achieved. In addition, the reference lists of all the included articles were screened for potentially relevant articles. The rationale for excluding studies was documented in the PRISMA diagram.

### Inclusion and exclusion criteria

Original, peer-reviewed studies that reported standardised, quantitative assessments of HR-QOL in adults (aged over 16) with a clinical or genetic diagnosis of OI were included in the review. Accepted studies were required to have either a control group, OI subgroup or reference population for comparison. Studies of children and adults were only included if HR-QOL outcomes for adults were reported separately. Studies that reported solely qualitative research methods (e.g. patient interviews) were not included. Searches were restricted to the English language. Studies classified as randomised controlled trials, observational studies (prospective or retrospective), cross-sectional studies, patient registry data and case series (with ten or more patients) were included. Single case reports, abstract-only papers, commentaries, study protocols and systematic reviews were excluded.

### Data extraction

Two reviewers extracted data independently from the accepted studies into Microsoft Excel. Data were collected under the following headings; study characteristics (design, country, date of publication), participant characteristics (age, sex, type of OI, number of patients), therapeutic intervention (if any), HR-QOL assessment tool used, HR-QOL outcome and determinants of HR-QOL. Accepted studies were referenced with a digital object identifier (DOI) to remove ambiguity from the study identity [20]. Discrepancies in the extracted data were resolved by reviewing the original study and reaching a consensus. Five authors were contacted for additional information during the data extraction process. The SF-36 is a frequently used, generic HR-QoL assessment that comprises four physical (physical function, bodily pain, role physical and general health) and four mental domains (vitality, social functioning, role limitations and mental health) [21]. Individual domains can be collated into a physical component score (PCS) and a mental component score (MCS). Scores are reported against a reference population with higher scores indicating better HR-QOL.

### Quality assessments

Standardised quality assessment tools from the National Heart, Lung and Blood Institute were used to determine the methodological quality and risk of bias of accepted studies [22]. Two investigators conducted these assessments independently and discussed any discrepancies before reaching a consensus. Link for quality assessment tools: https://www.nhlbi.nih.gov/health-topics/study-quality-assessment-tools

### Synthesis of evidence

We adopted a narrative summary approach when outlining study characteristics, patient demographics and primary and secondary study outcomes. Data were aggregated and then discussed by the authors until a consensus on the narrative outcomes was reached. A meta-analysis was not feasible given the heterogeneity in how results were reported; in addition, many studies were precluded from inclusion as SF-36 results were not reported with a mean and standard deviation/standard error. Including a small minority of the accepted studies in a meta-analysis would have introduced unacceptable levels of bias and unreliable results.

## Results

### Study selection and design

**Fig. 1:**
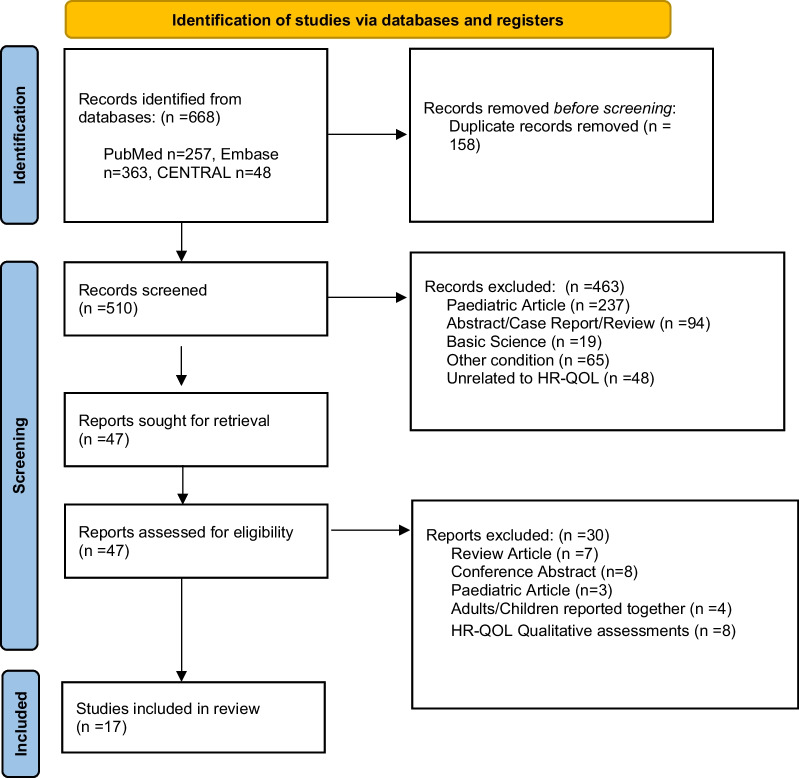
PRISMA diagram

A total of 668 articles were identified in the literature search; after duplicates were removed 510 articles underwent title and abstract screening. A total of 463 articles were excluded, leaving 47 articles to be analysed in the full-text review. Seventeen articles containing 1,648 adults with OI were accepted for inclusion in this review. Reasons for article exclusion were documented in the PRISMA diagram (Fig. 1). All accepted studies followed a cross-sectional design. The demographic details of the studies/ participants are recorded in Table 1. OI was compared to unmatched reference populations in thirteen studies, unmatched groups with X-linked hypophosphataemia (XLH) or fibrous dysplasia groups in two studies [23, 24] and matched controls in one study [25]. Another study compared two groups living with OI, depending on whether they had received bisphosphonates in childhood [26]. Thirteen studies classified OI type using the Sillence Classification, three studies used the terms ‘congenita/tarda’ [27–31] and one reported ‘more severe/less severe’ OI [26]. There was a wide geographic spread of study sites; nine European, six American, one Asian and one Australian.

**Table 1:** Demographic details of included studies

No	Author	Country	Study design	Study size	OI type	OI diagnosis	Sex	Age (years)
1	Orlando [35]	UK	Cross-sectional study	n = 78	Type 1– 32	Self-reported	F = 74%	43 ± 14.5^a^
					Type 3– 10		M = 26%	
					Type 4– 10			
					Unknown– 26			
2	Murali [36]	USA	Cross-sectional study	n = 320	Type 1– 173	Mixed clinical/ genetic	F = 67%	33.7 (25–47)^c^
					Type 3– 49		M = 33%	
					Type 4– 80			
					Type 5– 5			
					Unknown– 13			
3	Gjorup [24]	Denmark	Cross-sectional study	OI: n = 73	T 1– 56	Genetic	F = 55%	OI: 45.9^b^
					T 3/4– 17		M = 45%	
4	Yonko [37]	Multi-national	Cross-sectional study	n = 157	Type 1– 57	Self-reported	F = 69%	45.9 (19–81)^d^
					Type 3– 39		M = 30%,	
					Type 4– 33		Trans man = 1%	
					Unknown/ other- 28			
5	Matsushita [29]	Japan	Cross-sectional study	n = 40	Congenita– 13	Clinical	F = 55%	37.5^e^
					Tarda – 25		M = 45%	
					Unknown– 2			
6	Harsevoort [39]	Netherlands	Cross-sectional study	n = 99	Type 1– 72	N.R	F = 62%	45 (19–80)^d^
					Type 3– 13		M = 38%	
					Type 4– 14			
7	Khan [34]	USA	Cross-sectional study	n = 30	Type 1– 9	Genetic 53%	F = 80%	39 (19–67)^d^
					Type 3– 8	Clinical 47%	M = 20%	
					Type 4– 12			
					Bruck Syndrome– 1			
8	Gooijer [32]	Netherlands	Cross-sectional study	n = 322	Type 1– 220	Genetic	F = 59%	35.5 (27)^c^
					Type 3– 40		M = 41%	
					Type 4– 62			
9	Tosi [39]	USA	Cross-sectional study	n = 198	Adults not reported separately	Self-reported	F = 77%	Adults' age not reported separately
							M = 23%	
10	Feehan [26]	Australia	Cross-sectional study	n = 52	Less severe– 19	N.R	F = 54%	27.5 (23–36)^c^
					More severe– 33		M = 46%	
11	Arponen [25]	Finland	Cross-sectional study	n = 56	Type 1– 22	Self-reported	F = 73%	47 (16–75)^d^
					Type 3– 10		M = 27%	
					Type 4– 7			
					Unknown– 17			
12	Hald [31]	Denmark	Cross-sectional study	n = 84	Type 1– 58	Genetic	F = 55%	45 (18–78)^d^
					Type 3– 11		M = 45%	
					Type 4– 15			
13	Forestier-Zhang [23]	UK	Cross-sectional study	n = 43	Type 1- 18	N.R	F = 77%	40.4 ± 14.4^a^
					Type 3– 5		M = 23%	
					Type 4– 5			
					Unknown– 15			
14	Balkefors [33]	Sweden	Cross-sectional study	n = 29	Type 1 + 4– 29	N.R	F = 62%	41 (21–71)^d^
							M = 38%	
15	Nicolaou [30]	UK	Cross-sectional study	n = 22	Type 1– 7	N.R	F = 59%	24.7 (18–36)^d^
					Type 3– 10		M = 41%	
					Type 4– 5			
16	Widmann [28]	USA	Cross-sectional study	n = 30	Congenita– 18	Clinical	F = 70%	33 (20–50)^d^
					Tarda– 12		M = 30%	
17	Widmann [27]	USA	Cross-sectional study	n = 15	Congenita– 7	Clinical	F = 40%	33.3 (20–45)^d^
					Tarda– 8		M = 60%	

*N.R.* not reported, *OI* osteogenesis imperfecta, *XLH* X-linked hypophosphataemia

^a^Mean (standard deviation)

^b^Mean

^c^Median (interquartile range)

^d^Median (range)

^e^Median

### HR-QOL assessment used

The SF-36 was the most commonly used HR-QOL assessment tool, reported in 9 of 17 studies (Table 2) [26–34]. In addition, one study reported physical but not mental domains of the SF-36 [35] and another used an abbreviated version called the SF-12 [36]. There was significant heterogeneity in how the results of the SF-36 were reported. Three studies reported individual domains of the SF-36 [26, 30, 33], three reported PCS/MCS scores [27, 29, 34] and three reported both individual domains and PCS/ MCS scores [28, 31, 32]. Additionally, studies varied by reporting SF-36 results for the entire study cohort, by OI type or age category. There was a wide range of other HR-QOL assessments identified in the review. The St George’s Respiratory Questionnaire (SGRQ) [34, 37] and European Quality of Life 5 Dimensions 5 Level Version (EQ-5DL-5L) [23, 35] were each reported in two studies while all other assessments were reported once. These included non-specific HR-QOL assessments (World Health Organisation Quality of Life Assessment-WHOQOL-BREF [26]), functional assessments (Nottingham Extended Activities of Daily Living Scale-NEADL [35], International Physical Activity Questionnaire-IPAQ [26]), Patient Reported Outcomes Measurement Information System-PROMIS® scales [38] and assessments specific to oral HR-QOL (Oral Health Impact Profile-49-OHIP-49 [24]), life satisfaction (Li Sat-11 [33]) and fatigue (Fatigue Severity Scale-FSS [39], Functional Assessment of Chronic Illness Therapy –Fatigue-FACIT-F [35]). A description of all HR-QOL assessment tools included in the review is available in Additional file 2.

**Table 2:** HR-QOL outcomes

No	Author	HR-QOL Assessment(s)	Comparator	HR-QOL Outcome	Outcome by OI type	Associations with HR-QOL
1	Orlando [35]	PSC (SF-36)	Normative population	PCS ↓ *	PCS ↓ vs control for all OI types*	PCS correlated with fatigue (FACIT-F score), pain score, OI severity
				Physical function ↓ *	PCS ↓ T3/ 4 lower vs T1*	
				Bodily pain ↓ *		
				Role-physical ↓ *		
				General health ↓ *		
		European Quality of Life 5 Dimensions 5 Level Version (EQ-5DL-5L)	Normative population	EQ-5D-5L: Moderate/ severe difficulties	EQ-5D-5L: Self-care and severe mobility problems ↑ T3 OI*	N.R.
				Mobility 39% ↓ *		
				Usual activities 19% ↓ *		
				Pain 23% ↑ *		
				Self-care 19% ↔		
				Anxiety/ depression 12% ↔		
		Functional Assessment of Chronic Illness Therapy- fatigue (FACIT-F)	Normative population	FACIT-F: Fatigue ↑ in all OI types*	N.R.	N.R.
		Nottingham Extended Activities of Daily Living Scale (NEADL)		NEADL: T3 + 4 had severe problems with mobility and domestic tasks. T1 reported no/ mild difficulty performing most activities	NEADL: Difficulty was mobility and domestic tasks ↑ in T3 vs T1*	N.R.
2	Murali [36]	Short Form-12 (SF-12)	Normative population	PCS ↓ *	PCS ↓ in T3 vs T1/ 4*	N.R.
				MCS ↔	MCS ↑ in T3 vs norm population*	
					MCS in T1 and T4 ↔ vs norm population	
3	Gjorup [24]	Oral Health Impact Profile-49 (OHIP-49)	XLH group	OI lesser negative impact on oral-related QOL vs XLH *	T3/ 4 has ↓ oral QOL in 2 of 7 domains vs T1 (physical disability + handicap)*	N.R.
				Pain ↓*		
				Functional limitation ↓*		
				Psychological discomfort ↓*		
				Psychological disability ↓*		
				Handicap ↓*		
				Physical disability ↔		
				Social disability ↔		
4	Yonko [37]	St George's Respiratory Questionnaire (SGRQ)	Normative population	Respiratory related-QOL ↓*	Respiratory related-QOL↓ in T3/4 vs T1*	Respiratory related-QOL correlates with age, activity level and pulmonary/ cardiac co-morbidities*
						Scores do not correlate with degree of scoliosis
5	Matsushita [29]	Short Form-36 (SF-36)	Normative population	PCS ↓ 1st fracture < 2 yrs. old*	N.R.	↓ PCS associated with:
				PCS ↔ 1st fracture > 2yrs old		fracture < 2 yrs, > 5 lower extremity fractures, history of lower extremity surgery, shorter height, teeth abnormalities + cardio-pulmonary co-morbidities
				MCS ↔ vs control regardless of age at 1^st^ fracture		
6	Harsevoort [39]	Fatigue Severity Scale (FSS)	Normative populations (× 2)	Fatigue ↑ *	Fatigue independent OI type	N.R.
				Severe fatigue ↑ (27% vs 5%*)		
7	Khan [34]	SF-36	Normative population	PCS ↔		Pulmonary co-morbidities associated with ↓ MCS and PCS scores*
				MCS ↔		
		SGRQ		SGRQ: Respiratory -related QOL in OI ↓*	SGRQ: Respiratory -related QOL ↓in T3 vs T1*	SGRQ: Pulmonary co-morbidities associated with ↓ QOL*
						FEV1/FVC correlated with St George's QOL score*
8	Gooijer [32]	SF-36	Normative populations (× 2)	PCS ↓*	Physical function ↓ in T3 vs T1 + T4*	Bodily pain ↑ in older age group
				MCS ↔		
				Physical function ↓	Bodily pain ↑ in T1 + T4*	
				Bodily pain ↓		
				Role-physical ↓	Mental health, vitality ↓ in T1 only*	
				General health ↓		
				Vitality ↓ T1 only		
				Social functioning ↓ T1/3/4		
				Role limitations ↔		
				Mental health ↓ T1		
9	Tosi [39]	Patient-Reported outcomes Measurement Information System scales (PROMIS ®)	Normative PROMIS® population	General physical health ↓*	N.R.	N.R.
				Anxiety ↑*		
				Depression ↑*		
				Fatigue ↑*		
				Pain behaviour ↑*		
				Pain interference ↑*		
				Physical function ↓*		
				Satisfaction with social roles ↓*		
				Sleep disturbance ↑*		
10	Feehan [26]	SF-36	Bisphosphonate treatment in childhood vs	(i) SF-36: No difference between childhood treated and no treatment in childhood cohorts	Less severe forms of OI had improved physical functioning when treated in childhood*	N.R.
			(i) no treatment in childhood cohort	Physical function ↔		
			(ii) normative population	Bodily pain ↔		
				Role-physical ↔		
				General health ↔		
				Vitality ↔		
				Social functioning ↔		
				Role limitations ↔		
				Mental health ↔		
				(ii) Physical function, vitality and general health domains ↓ in childhood treated cohort vs norm population*		
		World Health Organisation Quality of Life Assessment (WHOQOL-BREF)	Bisphosphonate treatment in childhood vs	WHOQOL-BREF: No difference between childhood treated and no treatment in childhood cohorts	N.R.	N.R.
			(i) no treatment in childhood cohort	Physical ↔		
			(ii) healthy controls	Psychological ↔		
				Social relationships ↔		
				Environment ↔		
				Only physical domain ↓ vs healthy controls*		
		International Physical Activity Questionnaire (IAPQ)		IAPQ: higher physical activity in less severe OI who were treated in childhood*	N.R.	N.R.
11	Arponen [25]	Study specific fatigue, pain, sleep questionnaire	Control group	Fatigue 96% ↔	N.R.	Daily pain increased with age*
				Sleep disturbance 95% ↑*		Negative correlation between fatigue and OI severity*
				Daily pain 87% ↑*		
						Fatigue independent of OSA diagnosis
12	Hald [31]	SF-36	Normative population	PCS ↓*	PCS ↓ T3 vs T1/ 4*	↓ PCS scores correlate with OI severity and age*
				MCS ↔	MCS ↔ between OI types	↑ MCS scores correlate with ↑ education status*
				Physical function ↓*	MCS ↑ T3 vs norm population*	
				Bodily pain ↓*		
				Role-physical ↓*		
				General health ↓*		
				Vitality ↓* T1/ 4 only		
				Social functioning ↓* T1/ 3/ 4		
				Role limitations ↓ T1/ 4		
				Mental health ↔		
13	Forestier-Zhang [23]	EQ-5D-5L	FD, XLH groups	Severe/ extreme problems with:	N.R.	Age correlated with difficulty performing ADLs and worse perception of self-rated health
				Mobility 26%		
				Self-care 10%		
				Activities 17%		
				Pain 16%		
				Anxiety/ depression 7%		
				No difference between OI and XLH/FD		
14	Balkefors [33]	SF-36	Normative population	Physical function ↓*	N.R.	N.R.
				Bodily pain ↓*		
				Role-physical ↓*		
				General health ↓*		
				Vitality ↓*		
				Social functioning ↓*		
				Role limitations ↓*		
				Mental health ↓*		
		Life Satisfaction -11 (Li Sat-11)		High life satisfaction scores, lowest scores in physical health domain		
15	Nicolaou [30]	SF-36	Normative population	Physical function ↓*	N.R.	N.R.
				Bodily pain ↓*		
				Role-physical ↓*		
				General health ↓*		
				Vitality ↓*		
				Social functioning ↓*		
				Mental health ↔		
				Role limitations ↔		
16	Widmann [28]	SF-36	Normative population	PCS ↓*	N.R.	N.R.
				MCS ↔		
				Physical function ↓*		
				Bodily pain ↓*		
				Role-physical ↓*		
				General health ↔		
				Vitality ↔		
				Social functioning ↔		
				Role emotional ↔		
				Mental health ↔		
17	Widmann [27]	SF-36	Normative population	PCS ↓*	N.R.	Scoliosis, FEV1, VC, FVC correlate with PCS*
				MCS ↔		

*OI* osteogenesis imperfecta, *XLH* X-linked hypophosphataemia, T 1/3/4, type 1/3/4 OI, ↑ higher, ↓ lower, ↔ equal, * statistically significant, < less than, > greater than, *HR-QOL* health-related quality of life, *N.S*. not significant, *PCS* physical component score, *MCS* mental component score, *ADLs* activities of daily living, *FEV1* forced expiratory volume, *VC* vital capacity, *FVC* forced vital capacity, *OSA* obstructive sleep apnoea

### Impact of OI on HR-QOL

Physical HR-QOL was significantly reduced in adults with OI compared to reference populations. PCS or individual physical domain scores of the SF-36 were lower in eight of nine studies [35]. All six studies that measured bodily pain (SF-36 domain) reported significantly lower scores, reflecting a greater pain experience for individuals living with OI [28, 30–33, 35]. Arponen et al. also reported that 87% of people with OI experienced daily pain [25]. The impact of OI on HR-QOL is not limited to its skeletal manifestations. Two studies reported lower respiratory-related QOL as assessed by the St George's Respiratory Questionnaire [34, 37]. As a result of physical limitations, individuals experienced reduced mobility and difficulties with activities of daily living (ADLs) [40].

In terms of mental HR-QOL none of the six studies that reported MCS scores, identified any difference between the OI cohort and reference populations [27–29, 31, 32, 34]. A further five studies reported individual mental domains of the SF-36 [28, 30–33]. Four of these studies reported significantly lower vitality domains [30–33], four impaired social functioning [30–33], two impaired role limitations [31, 33], and two reduced mental health [32, 33] in at least one OI subtype or the entire cohort. Two of the three studies that included both individual mental domains and MCS scores, reported discordant results, with lower individual mental domains but overall preserved MCS scores [31, 32]. This indicates that the impact of a diagnosis of OI on mental HR-QOL is complex and is influenced by the particular domain assessed. In terms of mental health disorders, seven percent of adults reported severe to extreme levels of anxiety or depression as assessed by the EQ-5D-5L [23]. Another important aspect of HR-QOL in OI is fatigue. Orlando et al. reported higher rates of fatigue in patients living with OI, using the FACIT-F questionnaire [35]. The rate of severe fatigue was 42% in those with OI compared with 5% in the healthy population in another study [39]. Oral HR-QOL was the final aspect of HR-QOL evaluated in the review. A diagnosis of OI was found to exert less impact on oral HR-QOL than a diagnosis of XLH, as assessed by the OHIP-49 [24].

### Impact of OI type on HR-QOL

Physical HR-QOL was most impaired in those with type III OI. Four studies reported significantly lower PCS scores in individuals with type III OI compared with type I [31, 32, 35, 36]. Yonko et al. reported lower respiratory-related QOL with more frequent and severe respiratory symptoms experienced by those with type III OI compared to type I [37]. Overall, individuals with type III OI experienced additional challenges in everyday life including severe mobility issues and difficulty with self-care [35]. In addition, oral-related QOL was lower in two of seven domains of the OHIP-49 in those with type III and IV versus type I OI [24]. This finding is in keeping with the higher prevalence of dentinogenesis imperfecta in these types of OI. Despite its detrimental impact on physical, oral and respiratory-related QOL, no studies identified any differences in MCS or individual mental health domains between type III and type I OI. Hald et al. reported a higher MCS score in type III OI compared with the reference population, suggesting improved mental HR-QOL [31]. However, none of the four individual SF-36 mental domains for type III OI were higher while, the social functioning domain was significantly lower compared with the reference population. As we will discuss later, MCS scores should be interpreted with caution in individuals with severe physical impairments.

### Demographic and clinical associations with HR-QOL

Age had a negative association with multiple aspects of HR-QOL. Two studies reported that physical and respiratory-related QOL decreased with age [31, 37]. Individuals with OI were also more likely to experience pain and have greater difficulty undertaking their ADLs with increasing age [23, 25, 32]. Cardio-pulmonary status in OI was strongly associated with HR-QOL. The presence of cardio-pulmonary comorbidities was associated with impaired QOL in three studies, as assessed by PCS scores and the St George's Respiratory Questionnaire [29, 34, 37]. Khan et al. found that pulmonary function, measured by forced expiratory volume in one second/forced vital capacity (FEV1/FVC), correlated with respiratory-related QOL in the St George's Respiratory Questionnaire score [34]. This observation supported findings from an earlier study by Widmann et al. which demonstrated that lower PCS scores in OI correlated with the presence of scoliosis and impaired pulmonary function as measured by lower FEV1, vital capacity and FVC [27]. Two studies reported that female sex was associated with impaired aspects of HR-QOL. In one study, female sex was associated with lower respiratory-related QOL [37], while in another, women reported higher fatigue levels in one of the 9 FSS domains, namely, their work, family and social life [39]. Other factors associated with lower PCS scores included a first lower extremity fracture before 2 years, greater than 5 lower extremity fractures, a history of lower extremity surgery and dental abnormalities [29]. One study examined the impact of previous bisphosphonate treatment in childhood on adult HR-QOL. In a retrospective, cross-sectional study, those with mild OI, who were treated in childhood, had improved physical functioning (SF-36 domain) and higher levels of physical activity (IAPQ). However, there was no improvement in the overall PCS score or other domains of the SF-36 [26].

### Quality of evidence

Evaluation of study quality and risk of bias was performed using quality assessment tools from the National Heart, Lung, and Blood Institute. Of the 17 studies included in the review, 4 were rated as fair quality and 13 were rated as low quality. The full assessment of study quality is available in Additional file 3. Study quality was limited by their cross-sectional design and methodological reliance on surveys for data collection. Participant numbers were small, as expected as OI is an orphan condition. Furthermore, several studies relied on open calls to OI patients through websites and patient support networks which may impact the external validity with a selection bias to those linked with such groups [23, 25, 37].

## Discussion

This review collated results from 17 studies and found significant and wide-ranging impairments in HR-QOL in adults with OI. Individuals had significantly lower physical and respiratory HR-QOL and experienced higher rates of pain and fatigue. Although some deficits in mental HR-QOL were identified, results were generally comparable to the background population, while the prevalence of anxiety and depression was relatively low [23, 35]. These results support the findings of a previous systematic review by Dahan-Oliel et al. which reported reduced physical but preserved mental HR-QOL [19]. However, the conclusions for adults were based on three small studies with a total of 74 participants. Our current review is substantially larger, giving the results greater generalisability and also included aspects of HR-QOL including pain, fatigue, and oral health giving us a more detailed understanding of the impact of OI. These aspects of HR-QOL were previously identified as important outcome measures by the OI community [41].

There are numerous reasons why adults with OI have reduced physical HR-QOL. Although overall fracture frequency is highest in childhood, debilitating vertebral and hip fractures are more common in adulthood [42]. Musculoskeletal complications such as osteoarthritis also increase with age, leading to pain and stiffness which can limit physical function. Those diagnosed with type III OI, which is associated with the highest fracture risk, more severe bony deformities and the greatest physical disability, had lower physical HR-QOL compared to those with type I OI. Cardiopulmonary co-morbidities including sleep apnoea, restrictive lung defects and valvular abnormalities are common in OI and lead to dyspnoea, impaired sleep, fatigue and frequent respiratory tract infections [43–46]. A study of 157 participants found that adults with OI experienced more frequent and severe respiratory symptoms [37]. This limits aerobic capacity and exercise tolerance which contributes to difficulties with daily activities. Forestier-Zhang et al. reported severe or extreme mobility and self-care problems in 26% and 10% of adults with OI respectively [23]. Another barrier to physical activity is the fear of sustaining further fractures [47]. Engelbert et al. reported that reduced physical function was associated with lower physical HR-QOL [48]. Exercise has been shown to increase muscle strength, aerobic capacity and reduce fatigue in children with OI [49]. In adulthood, exercise is necessary to prevent functional decline and maintain independence. Therefore, physical activity, appropriate to an individual’s level of functioning should be advised as a potential means of improving physical HR-QOL. Concerning pulmonary co-morbidities, declining lung function is a leading concern among adults with OI [50, 51]. Addressing patients’ concerns over their respiratory status is an important component of a comprehensive OI assessment.

No studies reported lower MCS scores in individuals with OI compared with reference populations. However, the vitality and social functioning domains of the SF-36 were reduced in four out of five studies. Engelbert et al. noted that community participation was reduced in those with OI [48]. Despite the challenges posed by OI, mental health is relatively preserved. Only two of five studies reported reduced SF-36 individual mental health domains [32, 33] while rates of anxiety and depression, as assessed by the EQ-5D-5L were also relatively low [23, 35]. One reason for this may be the development of coping skills in childhood in response to physical challenges. Ablon et al. reported that resilience was a common trait in adults with OI [52]. Families also emphasise education over physical pursuits in childhood. As a result, high levels of educational achievement are common in the OI community and help to give a sense of satisfaction despite physical or social limitations [33, 50, 53]. Hald et al. showed that higher education levels correlated with improved MCS scores, suggesting education has a beneficial effect on mental HR-QOL [31].

Discordant results between MCS and individual mental domain scores in this OI review highlight the need to interpret SF-36 summary scores with care  [54]. Calculation of PCS and MCS scores assumes a zero correlation between physical and mental HR-QOL. To generate this, MCS scores are calculated by giving negative weighting to physical domains and positive weighting to mental domains [55]. This can result in an overestimation of MCS scores in those patients with severe physical impairments. For instance, Hald et al. reported a significantly higher MCS score in type III OI compared with healthy controls ^(231)^. The MCS score was higher despite none of the individual mental domains being higher, while the social functioning domain was significantly lower (see Table 2). Overestimation of MCS scores has been demonstrated in other conditions with physical limitations, including multiple sclerosis and orthopaedic trauma [56, 57]. Studies of individuals with OI that report PCS/MCS without individual domain scores may not identify impairments in specific mental domains. Given the significant heterogeneity in reporting SF-36 outcomes that this review identified, there is a clear need to standardise reporting in future. Comprehensive reporting requires publishing individual domain and PCS/MCS scores with standard deviations for the entire cohort [54]. Additional results can also be categorised by age or OI type depending on the study size and design.

The authors reviewed the literature originally to inform the selection of HR-QOL measures for our rare bone clinic when undergoing assessment as a European Reference Network Centre for rare bone disease and undertook this systematic review when the limitations of the available literature became clear. A consensus on the HR-QOL assessment tool or group of tools that should be used in clinical and research settings is required to standardise reporting of patient outcomes. As this review demonstrates, generic assessments of HR-QOL have been widely used. We need to be cognisant that clinician-reported outcomes may not be meaningful endpoints for patients. Generic questionnaires fall short of capturing the specific experience of living with OI. Involving patients in the development of PROs and disease-specific questionnaires helps to align the clinician and patient in determining important quality-of-life measures for patients with OI. In recent years, several studies have identified PROs chosen by the OI community [41, 50, 51]. Although this has improved our understanding of the OI communities’ values and concerns, a consensus on which PROs should be used has yet to be reached. Further collaboration between all stakeholders including the OI community, clinicians, nurse specialists, physiotherapists and researchers is necessary to achieve this goal. Patient-focused outcomes should be measured in all rare disease research studies, including OI. A trial examining the efficacy of burosumab improved our understanding of XLH when it reported HR-QOL as a secondary endpoint [58]. It is encouraging to see the TOPaZ trial, which is currently investigating the efficacy of teriparatide followed by zoledronic acid in adults with OI, has also included HR-QOL and functional assessments in its endpoints ^(59)^. Another area requiring further research is how HR-QOL changes over time. Age was associated with worsening HR-QOL in two studies in our review [31, 37]. However, the studies in this review assessed HR-QOL at a single point in time and therefore did not take into account recent events such as a fracture or hospitalisation. The Brittle Bone Disorders Consortium is currently enrolling participants in a longitudinal study [60]. Over time, studies such as these should help us understand how the burden of OI evolves over an individual’s lifetime and identify factors that either improve or worsen HR-QOL.

This is the first systematic review to focus on the impact of OI on adults’ HR-QOL. The inclusion of seventeen studies constitutes a large and comprehensive review of the literature given the rarity of this condition. It is a timely summary of the current evidence base, as eight of the seventeen studies have been published since 2020. Another strength is the wide geographic spread of studies from four continents, meaning the conclusions apply to a wide audience. The review has several limitations including the generally low quality of evidence available. Sources of potential bias included selection bias, study outcomes being compared to populations that were not age- or sex-matched and patients self-reporting their OI type in some studies. Most studies in the review were single-centre and contained small numbers of participants. These studies were underpowered to identify small differences with reference populations and within OI groups. Multi-centre studies or large patient registries are required to build on the current evidence base. Although this review identified significant differences in HR-QOL between types of OI, individualised clinical assessments are essential given the heterogeneity of this disorder. It is important to consider that the phenotype is not solely dependent on the type of OI or even the affected gene but also depends on the location and specific nature of the gene mutation [61]. Despite these limitations, the review provides a comprehensive analysis of the current literature and expands our current understanding of the impact of OI on HR-QOL in adults.

## Conclusion

This is the first systematic review to assess HR-QOL exclusively in adults with OI. Physical and respiratory HR-QOL was reduced while fatigue and pain were more prevalent in adults with OI. Mental HR-QOL was relatively preserved in individuals with OI, although some deficits were reported. It is important to interpret MCS scores with the corresponding individual mental domain scores to identify deficits in mental HR-QOL. Physical and respiratory but not mental HR-QOL was reduced in individuals with type III compared with type I OI. Increasing age and the presence of cardio-pulmonary co-morbidities were associated with lower HR-QOL.

## Supplementary Information

**Additional file 1**. Search strategy.

**Additional file 2**. HR-QOL assessments.

**Additional file 3**. Risk of Bias assessment.

## Data Availability

Data available on request to primary author.

## Abbreviations

HR-QOL: Health-related quality of life
OI: Osteogenesis Imperfecta
XLH: X-linked hypophosphataemia
FD: Fibrous Dysplasia
SF-36: Short Form-36
PCS: Physical Component Score
MCS: Mental Component Score
