# Supplementary material for: Systematic review of health related-quality of life in adults with osteogenesis imperfecta
Source: Orphanet J Rare Dis. 2023 Feb 22;18:36. doi: 10.1186/s13023-023-02643-3 (PMC9945612; doi:10.1186/s13023-023-02643-3)
Supplement: Supplementary file 1 — Additional file 1. Search strategy. [file 13023_2023_2643_MOESM1_ESM.docx]

| **Embase** | | |
| --- | --- | --- |
|  |  |  |
| 1 | 'quality of life'/exp OR 'quality of life' | 694,125 |
|  |  |  |
| 2 | 'daily life activity'/exp OR 'daily life activity' | 101,749 |
|  |  |  |
| 3 | 'health status'/exp OR 'health status' | 310,295 |
|  |  |  |
| 4 | 'patient-reported outcome'/exp OR 'patient-reported outcome' | 45,477 |
|  |  |  |
| 5 | 'disease burden'/exp OR 'disease burden' | 86,433 |
|  |  |  |
| 6 | 'health assessment questionnaire'/exp OR 'health assessment questionnaire' | 13,405 |
|  |  |  |
| 7 | #1 OR #2 OR #3 OR #4 OR #5 OR #6 | 1,095,190 |
|  |  |  |
| 8 | 'osteogenesis imperfecta'/exp OR 'osteogenesis imperfecta' OR (('osteogenesis'/exp OR osteogenesis) AND imperfecta) | 9,011 |
|  |  |  |
| 9 | #7 AND #8 | **363** |
|  |  |  |
| **CENTRAL** | | |
|  |  |  |
| 10 | osteogenesis imperfecta* | 144 |
|  |  |  |
| 11 | quality of life | 149,720 |
|  |  |  |
| 12 | activities of daily living | 14,558 |
|  |  |  |
| 13 | health status | 66,478 |
|  |  |  |
| 14 | patient reported outcome | 45,782 |
|  |  |  |
| 15 | disease burden | 15,093 |
|  |  |  |
| 16 | health impact assessment | 23,315 |
|  |  |  |
| 17 | ((osteogenesis imperfecta) AND (quality of life OR activities of daily living OR health status OR patient reported outcome OR disease burden OR health impact assessment)) | **48** |
|  |  |  |
| **Pubmed** | | |
|  |  |  |
| 18 | ("Osteogenesis Imperfecta"[Mesh]) OR (osteogenesis imperfecta) | 6,139 |
|  |  |  |
| 19 | ("Quality of Life"[Mesh]) OR (quality of life) | 477,363 |
|  |  |  |
| 20 | ("Activities of Daily Living"[Mesh]) OR (activities of daily living) | 131,661 |
|  |  |  |
| 21 | ("Quality-Adjusted Life Years"[Mesh]) OR (quality adjusted life year) | 24,274 |
|  |  |  |
| 22 | ("Health Status"[Mesh]) OR (health status) | 794,568 |
|  |  |  |
| 23 | ("Cost of Illness"[Mesh]) OR (disease burden) | 163,251 |
|  |  |  |
| 24 | (health assessment questionnaire) | 356,124 |
|  |  |  |
| 25 | ("Patient Reported Outcome Measures"[Mesh]) OR (patient reported outcomes) | 106,694 |
|  |  |  |
| 26 | (((((((((((((quality of life) OR ("Quality of Life"[Mesh])) OR ("Activities of Daily Living"[Mesh])) OR (activities of daily living)) OR ("Quality-Adjusted Life Years"[Mesh])) OR (quality adjusted life year)) OR ("Health Status"[Mesh])) OR (health status)) OR ("Cost of Illness"[Mesh])) OR (disease burden)) OR (health assessment questionnaire)) OR ("Patient Reported Outcome Measures"[Mesh])) OR (patient reported outcomes)) AND ((osteogenesis imperfecta) OR ("Osteogenesis Imperfecta"[Mesh])) | **257** |
|  |  |  |
| **Total number of articles identifies (including duplicates)** | | **668** |
