# Supplementary material for: Systematic review of health related-quality of life in adults with osteogenesis imperfecta
Source: Orphanet J Rare Dis. 2023 Feb 22;18:36. doi: 10.1186/s13023-023-02643-3 (PMC9945612; doi:10.1186/s13023-023-02643-3)
Supplement: Supplementary file 2 — Additional file 2. HR-QOL assessments. [file 13023_2023_2643_MOESM2_ESM.docx]

**HR-QoL Assessment tools**

**Short Form-36 (SF -36)**

The SF-36 is a widely used, generic, self-reported health assessment tool. It comprises four physical (physical function, role physical, role emotional and social function) and four mental domains (pain, mental health, vitality and general health). Individual domains can then be collated into a physical component score (PCS) and mental component score (MCS). These scores are reported against a normative population, higher scores indicate a better HR-QOL.

**Short Form-12 (SF-12)**

The SF-12 is an abbreviated form of the SF-36. The questionnaire consists of 12 questions covering eight identical domains to the SF-36. It was designed to lessen the burden on respondents.

**St George’s Respiratory Questionnaire**

The St George’s Respiratory Questionnaire was designed to assess the impact of respiratory disorders on HR-QOL. The questionnaire consists of three categories; symptom frequency and severity, activities that cause breathlessness and impact on daily life. Symptoms examined include dyspnoea, cough, sputum production and wheezing. The questionnaire is scored from 0 -100, with higher scores reflecting greater limitations.

**Oral Health Impact Profile-49 (OHIP-49)**

The OHIP-49 is an oral HR-QOL assessment tool which measures the impact of oral disorders on people’s well-being. It consists of seven domains, namely, functional limitation, physical pain, psychological discomfort, physical disability, psychological disability, social disability and handicap. Individuals must answer how frequently they are affected (never, hardly ever, occasionally, fairly often or very often) for each of the 49 questions.

**European Quality of Life 5-Dimension 5-Level Version (EQ-5DL-5L)**

The EQ-5DL-5L is a generic HR-QOL assessment tool. Individuals are asked to rate their difficulty in five areas; mobility, self-care, usual activities, pain/discomfort and anxiety/depression. The level of difficulty is rated on a five point scale ranging from no problems to extreme problems. The EQ-5DL-5L also contains a visual analogue scale. Individuals rate their health along a scale with endpoints that are labelled ‘The best health you can imagine’ and ‘The worst health you can imagine’.

**Fatigue Severity Scale (FSS)**

The FSS is a nine question assessment tool used to quantify the degree of fatigue experienced by individuals with chronic conditions. It addresses the impact of fatigue on motivation, physical functioning, duties and work, family and social life.

**Nottingham Extended Activities of Daily Living Scale (NEADL)**

The NEADS assesses four categories of daily activities: mobility, kitchen activities, domestics and leisure activities. Individuals are asked to record if they have undertaken 21 specific tasks in recent weeks (e.g.- climbing stairs) and with what level of difficulty they encountered. ~~(on your own, own your own with difficulty, with help and not at all).~~

**Functional assessment of chronic illness therapy – fatigue (FACIT-F)**

This is a 13-item questionnaire that assesses the severity of fatigue during routine daily activities. The level of fatigue is measured on a 5 point scale (not at all, a little, somewhat, quite a bit, very much). Higher scores indicate more severe fatigue.

**World Health Organisation Quality of Life (WHOQOL-BREF)**

This questionnaire assesses HR-QOL in four domains; physical health, psychological wellbeing, social relationships and environment. It is an abbreviated form of the WHOQOL-100. It contains 26 questions, each of which are rated on a five point scale.

**Patient-Reported Outcomes Measurement Information System (PROMIS®)**

Series of standardised scales to assess patient’s physical, mental and social wellbeing. Examples of scales include pain intensity, fatigue, sleep disturbance, anxiety, depression and ability to participate in social roles and activities. PROMIS scales incorporate computer adaptive testing, allowing the precise measurement of health status within a few questions.

**International Physical Activity Questionnaire (IPAQ)**

**Assessment** of level of physical activity undertaken. Covers 27 questions over 5 domains; job, transportation, housework, recreation and time spent sitting. Individuals can be classified as having low, moderate or high levels of physical activity.
